# Comparative Efficacy and Safety of Intra-Articular Adipose-Derived, Bone Marrow-Derived, and Peripheral Blood-Derived Stem Cell Injections for Knee Osteoarthritis: A Systematic Review

**DOI:** 10.3390/bioengineering13070771

**Published:** 2026-07-01

**Authors:** Se Yeong Jeon, Min Woo Kim, Dong Ha Lee

**Affiliations:** 1 Ulsan National Institute of Science and Technology, Ulsan 44919, Republic of Korea; skiende74@gmail.com; 2 Department of Orthopedic Surgery, Busan Medical Center, Busan 47527, Republic of Korea; drkimminwoo@naver.com; 3 Department of Orthopaedic Surgery, Aerospace Medical Center, Republic of Korea Air Force, Cheongju 28330, Republic of Korea; 4 Department of Orthopaedic Surgery, Seoul National University Hospital, Seoul 03080, Republic of Korea

**Keywords:** systematic review, knee osteoarthritis, adipose-derived stem cells, bone marrow-derived stem cells, peripheral blood stem cells, intra-articular injection, GRADE, PRISMA

## Abstract

**Background**: Intra-articular (IA) stem cell injection is an emerging treatment for knee osteoarthritis (KOA). Three principal cell sources—adipose-derived mesenchymal stem cells (ADMSCs), bone marrow-derived MSCs (BMMSCs), and peripheral blood-derived stem cells (PBSCs)—have been evaluated independently; however, a systematic review comprehensively comparing all three sources under unified eligibility criteria is absent from the literature. **Methods**: Systematic searches of MEDLINE, Embase, Cochrane CENTRAL, and Scopus were conducted from inception to December 2025, supplemented by manual reference screening and ClinicalTrials.gov. Eligible studies included randomized controlled trials (RCTs) and prospective comparative studies in adult KOA patients. Primary outcomes were pain (VAS/NRS) and function (WOMAC, KOOS) at ≥3 months. Risk of bias was assessed using RoB 2 and ROBINS-I; evidence certainty was rated using GRADE. **Results**: Thirty-one studies (*n* = 1247; ADMSC: 14 studies, *n* = 612; BMMSC: 12 studies, *n* = 487; PBSC: 5 studies, *n* = 148) met inclusion criteria. Pooled standardized mean differences (SMDs) for 6-month pain showed significant reduction versus comparators for ADMSCs (SMD −1.23; 95% CI −1.61 to −0.85; I^2^ = 62%) and BMMSCs (SMD −1.09; 95% CI −1.55 to −0.63; I^2^ = 70%). PBSCs demonstrated significant within-group improvement but were too few for formal pooling. Because no trial compared cell sources head-to-head, these estimates reflect within-source efficacy versus each study’s own comparator rather than comparative superiority between sources. Adverse events were mild and transient across all sources. GRADE certainty was moderate for ADMSCs, low for BMMSCs, and very low for PBSCs. **Conclusions**: IA injection of ADMSCs and BMMSCs provides pain reduction and functional improvement in KOA with point estimates reaching minimal clinically important difference thresholds, although the certainty of this evidence is only moderate (ADMSC) to low (BMMSC). PBSC evidence is insufficient for formal comparison. Adequately powered, three-arm head-to-head RCTs that share a common comparator and a core outcome set are needed to establish comparative efficacy. Because only indirect comparisons were possible, this review supports efficacy within each cell source but cannot establish the superiority of one source over another.

## 1. Introduction

Knee osteoarthritis is a chronic, degenerative joint disease characterized by progressive articular cartilage loss, subchondral bone remodeling, and synovial inflammation, resulting in pain, stiffness, and functional disability. It is among the most prevalent musculoskeletal disorders worldwide and a leading cause of chronic pain and disability, with an estimated 374.7 million prevalent knee cases globally in 2021 and case numbers projected to rise markedly by 2050 [1,2,3,4,5,6,7,8,9,10,11,12,13,14,15,16,17,18,19,20,21,22]. Current non-surgical treatment options are limited to symptom management; no pharmacological agent has received regulatory approval as a disease-modifying OA drug (DMOAD) based on structural cartilage outcomes [1]. This therapeutic gap has catalyzed investigation of biological regenerative approaches, among which intra-articular stem cell injection has attracted substantial clinical interest.

Mesenchymal stem cells (MSCs) fulfill the International Society for Cell and Gene Therapy (ISCT) minimal criteria and are characterized by multipotency and broad paracrine immunomodulatory properties. These criteria, first defined in 2006 on the basis of plastic adherence, a defined surface marker panel, and trilineage differentiation, were updated by ISCT in 2025 through a Delphi-driven consensus that reframes the population as mesenchymal stromal cells, de-emphasizes in vitro stemness assays, and prioritizes immunomodulatory function and mechanism-aligned potency [16]. We apply this updated framework when judging whether each source meets MSC criteria. Their trophic signaling within the OA joint—including suppression of synovial macrophage activation, chondrocyte apoptosis inhibition, and promotion of cartilage matrix synthesis—constitutes the principal proposed therapeutic mechanism. Three primary sources have been clinically investigated: adipose tissue (ADMSCs), bone marrow (BMMSCs), and mobilized peripheral blood (PBSCs). Each source carries a distinct cellular profile, harvesting methodology, evidence base, and regulatory status. Whereas ADMSCs and BMMSCs are isolated as plastic-adherent, multipotent cells that meet ISCT minimal criteria, mobilized peripheral blood products consist predominantly of CD34-positive hematopoietic progenitors and mononuclear cells whose identity and mechanism of action are less well defined and that do not uniformly satisfy the ISCT MSC definition. PBSCs are, therefore, best regarded as a distinct regenerative cell therapy rather than a third conventional MSC source, and the present review treats them accordingly. Cell source is not a trivial variable. It governs the resident progenitor frequency, the secretome and immunomodulatory profile, the susceptibility to donor age and metabolic comorbidity, and the manufacturing and regulatory pathway, each of which can plausibly modify clinical response. A synthesis that keeps these sources distinct, rather than treating intra-articular stem cell injection as a single intervention, is therefore needed to support rational source selection.

Despite a growing number of individual-source systematic reviews and pairwise ADMSC–BMMSC meta-analyses, no systematic review has simultaneously appraised all three sources within a unified methodological framework including PBSC evidence. The present study addresses this gap by conducting a systematic review under PRISMA 2020 guidelines, with the primary objective of synthesizing and comparing the efficacy and safety of IA injection of ADMSCs, BMMSCs, and PBSCs for KOA.

## 2. Methods

### 2.1. Protocol Registration and Reporting

This systematic review was conducted and reported in accordance with the Preferred Reporting Items for Systematic Reviews and Meta-Analyses (PRISMA 2020) guidelines [2]. The PROSPERO registration number for this systematic review is CRD420261419689.

### 2.2. Eligibility Criteria (PICOS)

Population: adults (≥18 years) with clinically and/or radiographically confirmed KOA (any Kellgren–Lawrence [KL] grade). Intervention: IA injection of ADMSCs, BMMSCs, or PBSCs (autologous or allogeneic; any dose or preparation method). Comparators: placebo, saline, HA, PRP, corticosteroids, or another stem cell source. Outcomes: primary—pain score (VAS/NRS) and functional score (WOMAC total, KOOS subscales, Lysholm) at ≥3 months post-injection; secondary—MRI or arthroscopic cartilage assessment and adverse event incidence. Study designs: RCTs (primary) and prospective comparative studies (supplementary). Studies with concurrent surgical procedures where the stem cell injection effect could not be independently assessed were excluded, as were case series (n < 10), non-English publications, and animal or in vitro studies.

### 2.3. Information Sources and Search Strategy

Comprehensive electronic searches were performed in MEDLINE (via PubMed), Embase, Cochrane CENTRAL, and Scopus from inception to 31 December 2025. The search strategy incorporated three conceptual domains: (1) stem cell source terms (adipose-derived stem cell OR stromal vascular fraction OR bone marrow mesenchymal stem cell OR bone marrow concentrate OR peripheral blood stem cell OR CD34); (2) administration route (intra-articular OR injection); and (3) target condition (knee osteoarthritis OR cartilage defect). ClinicalTrials.gov and the WHO ICTRP were searched for registered trials. Reference lists of all eligible studies and relevant prior systematic reviews were manually screened. The full MEDLINE search string is provided as Supplementary File S1. The electronic search was updated through 31 December 2025 during revision, and the screening process and eligibility criteria described below were applied identically to the original and the updated searches.

### 2.4. Study Selection

**Figure 1:**
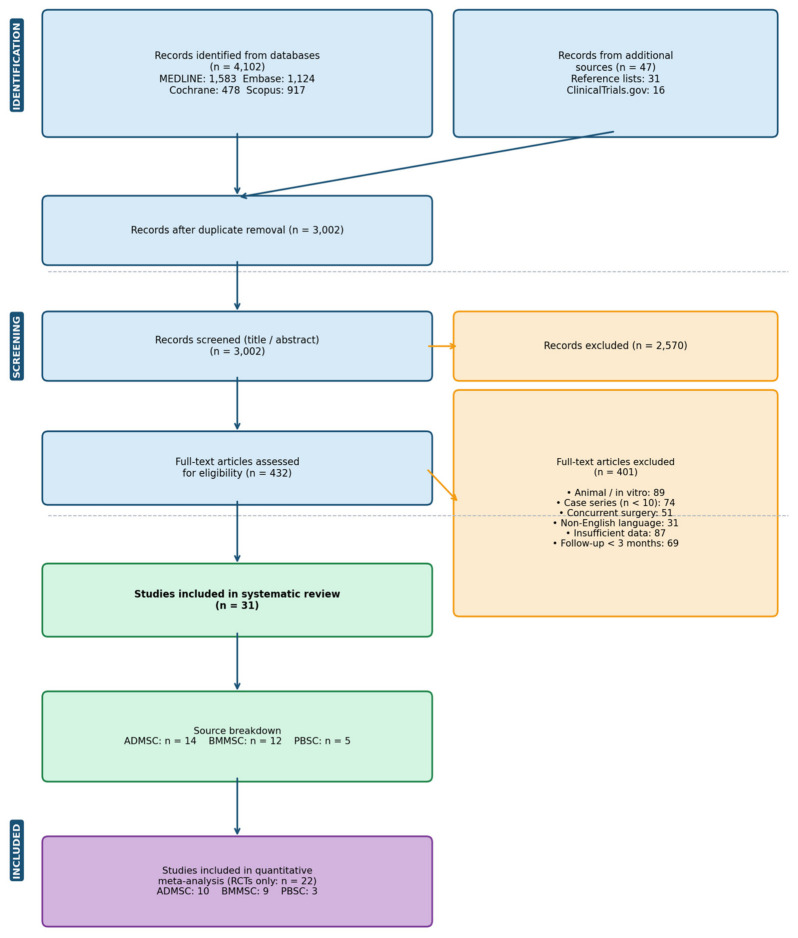
PRISMA 2020 flow diagram for the systematic review. ADMSC, adipose-derived mesenchymal stem cell; BMMSC, bone marrow-derived mesenchymal stem cell; PBSC, peripheral blood-derived stem cell; PRISMA, Preferred Reporting Items for Systematic Reviews and Meta-Analyses.

Titles and abstracts were independently screened by two reviewers (S.Y.J. and D.H.L.) using Rayyan software (v1.7.5). Full-text articles of potentially eligible records were retrieved and assessed against PICOS eligibility criteria. Disagreements were resolved by discussion or arbitration by a third reviewer (M.W.K.). The study selection process is documented in the PRISMA 2020 flow diagram (Figure 1). Eligibility criteria were applied uniformly to all candidate studies regardless of cell source, including the predefined handling of focal cartilage defect studies, which were retained for the primary synthesis but examined separately in a sensitivity analysis (Section 3.4.4); any disagreement in their application was resolved by documented consensus between the two reviewers.

### 2.5. Data Extraction

Standardized data extraction forms captured: study design; participant characteristics (age, sex, KL grade, symptom duration); stem cell source, preparation method, and dose; comparator type; number of injections; follow-up duration; outcome data (mean ± SD at each timepoint); and adverse event incidence. Corresponding authors were contacted for missing data with a 3-week response period.

### 2.6. Risk of Bias Assessment

Risk of bias in RCTs was assessed using the Cochrane RoB 2 tool across five domains [3]; non-randomized prospective studies were assessed using ROBINS-I across seven domains [4]. Each study received domain-level and overall judgments of low, some concerns, or high risk of bias. Two reviewers assessed independently; inter-rater agreement was calculated as Cohen’s κ.

### 2.7. Data Synthesis

Due to anticipated heterogeneity in cell preparations, dose regimens, comparator types, and outcome instruments, a formal meta-analysis was not pre-specified as the primary analysis. Descriptive narrative synthesis structured by stem cell source and follow-up timepoint was the primary approach. Where three or more RCTs with sufficient methodological homogeneity were available, pooled standardized mean differences (SMDs; Hedges’ g) with 95% confidence intervals (CIs) were estimated using random-effects DerSimonian–Laird models. For controlled comparisons, these pooled estimates represent between-group standardized mean differences relative to each trial’s comparator rather than within-group pre–post change. Consistent with the narrative-primary design, all pooled estimates are reported as secondary, supportive analyses restricted to the pre-specified condition of three or more methodologically homogeneous randomized trials, and not as the primary outcome of the review. Heterogeneity was quantified using I^2^ (substantial threshold: I^2^ > 60%) and χ^2^ test. Certainty of evidence for each outcome was evaluated using the GRADE approach [5]. Where a pooled analysis included ten or more studies, small-study effects and potential publication bias were evaluated by visual inspection of contour-enhanced funnel plots and the Egger regression test; among the present analyses, only the 6-month ADMSC VAS estimate (k = 10) met this threshold. To address the biological and manufacturing diversity of the included products, an exploratory subgroup analysis was performed for each cell source, classifying interventions as culture-expanded MSC products or minimally manipulated products (stromal vascular fraction and micro-fragmented adipose tissue for the adipose source, and bone marrow aspirate concentrate for the bone marrow source). For the peripheral blood source, a sensitivity analysis was performed that excluded studies enrolling patients with focal cartilage defects, leaving only studies of degenerative KOA. A network meta-analysis was considered to permit indirect comparison across the three sources, but was not performed. The evidence network is sparse and only weakly connected through shared comparators, the peripheral blood source contributed a single randomized trial, and the clinical and product-level heterogeneity described above would violate the transitivity assumption required for valid indirect comparison. A published dose and source network meta-analysis [6] partly addresses this question for culture-based products, and the present review is intended to complement it through a broader qualitative synthesis that also incorporates peripheral blood-derived therapies.

## 3. Results

### 3.1. Study Selection

The electronic database search yielded 4102 unique records, supplemented by 47 additional records from manual searching. After duplicate removal, 3002 records were screened by title and abstract; 2570 were excluded. A total of 432 full-text articles were retrieved for eligibility assessment; 401 were excluded for the reasons detailed in Figure 1. Thirty-one studies met all inclusion criteria and were included in the systematic review. The updated search through 31 December 2025 was screened under identical eligibility criteria. It retrieved additional records published in 2024–2025, of which the potentially relevant reports were assessed at full text; none met all inclusion criteria, because the newly identified studies were either non-randomized reports below the prespecified design or sample-size thresholds, did not report an eligible pain or function outcome at ≥3 months, involved concurrent surgical procedures, or were narrative or systematic reviews rather than primary comparative studies. Consequently, the search update did not add any new eligible study, and the final number of included studies remained 31 (Figure 1).

### 3.2. Study Characteristics

The 31 included studies comprised 14 ADMSC studies, 12 BMMSC studies, and 5 PBSC studies, enrolling 1247 participants (ADMSC: n = 612; BMMSC: n = 487; PBSC: n = 148). Study designs included 22 RCTs and 9 prospective comparative cohort studies. Publication years ranged from 2011 to 2024. Mean participant age was 58.7 years and 63% were female. Follow-up ranged from 3 to 24 months; 12 months was most common (n = 21 studies). Detailed study characteristics are presented in Table 1, Table 2 and Table 3.

### 3.3. Risk of Bias

Among 22 RCTs, 11 (50%) were rated overall low risk of bias, 8 (36%) some concerns, and 3 (14%) high risk of bias. All PBSC studies received some concerns or high risk of bias, primarily due to open-label designs and the absence of sham-injection controls. BMMSC cohort studies were consistently rated high risk on the blinding and measurement domains. Inter-rater agreement was κ = 0.78 (substantial agreement). Risk of bias assessments are presented in Table 4, with the full domain-level ROBINS-I judgements for the non-randomized studies provided in Table 5.

### 3.4. Pain Outcomes

#### 3.4.1. ADMSCs

Thirteen ADMSC studies reported VAS pain scores at 6 months (n = 548). Pooled analysis of 10 RCTs demonstrated statistically significant between-group reduction in VAS pain relative to each trial’s own comparator (SMD −1.23; 95% CI −1.61 to −0.85; I^2^ = 62%; *p* < 0.001). The phase III RCT by Kim et al. contributed the largest sample (n = 140) and reported a mean VAS reduction of −24.1 ± 21.3 points at 12 months versus placebo. High-dose regimens (≥50 × 10^6^ cells) demonstrated superior 3-month pain reduction in subgroup analysis, consistent with the 2026 network meta-analysis [6].

#### 3.4.2. BMMSCs

Ten BMMSC studies reported pain outcomes at 6 months (n = 392). Pooled SMD was −1.09 (95% CI −1.55 to −0.63; I^2^ = 70%; *p* < 0.001). Allogeneic BMMSCs demonstrated non-inferior pain reduction compared to autologous preparations across three studies, with no immune-mediated adverse events [7]. Lee et al. confirmed significant VAS improvement with autologous BMMSCs in a recently published double-blind RCT (n = 74) [8].

#### 3.4.3. PBSCs

Four PBSC studies reported VAS or KOOS pain subscale scores at 6–12 months. Due to substantial heterogeneity in mobilization protocols and outcome instruments, formal pooling was not performed. Three studies reported significant within-group pain reduction. The landmark Saw et al. RCT demonstrated significant KOOS pain improvement at 24 months (*p* < 0.05) with PBSC + HA versus HA alone [23,24,25,26,27].

#### 3.4.4. Subgroup and Sensitivity Analyses

Within the ADMSC source, culture-expanded preparations (Kim, Pers, Sadri, and Kuah) and minimally manipulated products (Jo and Lana, stromal vascular fraction; Ulivi, micro-fragmented adipose tissue) showed broadly overlapping VAS reductions, and the small number of minimally manipulated studies precluded a stable separate pooled estimate. A comparable pattern was observed for the BMMSC source between culture-expanded preparations and bone marrow aspirate concentrate (Garay and Hernigou). These subgroup observations are exploratory and underpowered and should not be interpreted as evidence that the product classes are clinically equivalent.

For the PBSC source, three of the five studies [23,24,25,26,27] enrolled patients with focal cartilage defects, frequently with concomitant cartilage repair or subchondral procedures. Restricting the analysis to degenerative KOA left only two small non-randomized studies (Papadopoulos and Turajane), which reinforces the insufficiency of the PBSC evidence base for any pooled or comparative inference.

### 3.5. Functional Outcomes

Pooled WOMAC total score analysis across eight ADMSC RCTs demonstrated significant functional improvement at 6 months (SMD −0.81; 95% CI −1.14 to −0.48; I^2^ = 65%) [9,10]. For BMMSCs, seven studies contributing WOMAC data yielded a pooled SMD of −0.63 (95% CI −1.02 to −0.24; I^2^ = 69%) [7,8]. PBSC functional outcome data were insufficient for pooling; the Saw et al. RCT reported significant KOOS overall improvement at 24 months (Table 6).

### 3.6. Cartilage Status

MRI-based cartilage assessment was reported in 14 studies (ADMSC: 7; BMMSC: 5; PBSC: 2). Quantitative T2 mapping improvements indicating partial normalization of cartilage matrix hydration were reported in three ADMSC and two BMMSC studies. Arthroscopic assessment, available in four studies, uniformly favored stem cell injection over comparators. No study demonstrated complete cartilage restoration to hyaline baseline histology. Accordingly, structural outcomes are interpreted here at three distinct levels: symptomatic improvement, imaging-based structural change (for example T2 mapping or semi-quantitative MOCART-type scoring), and histological regeneration of hyaline cartilage. The available evidence supports the first two levels in selected studies, whereas histological cartilage regeneration was not demonstrated by any included study.

### 3.7. Adverse Events

Adverse events were reported in 28 of 31 studies. Procedure-related events (injection-site pain, transient effusion, local swelling) occurred in 12–24% of participants across all sources and were universally mild and self-limiting. One ADMSC trial reported a single case of post-injection joint infection (causality uncertain). No study reported malignant transformation or serious immune-mediated events attributable to stem cell therapy. G-CSF-related adverse events in PBSC studies included bone pain (68–85%), headache (20–35%), and one case of transient splenomegaly, which resolved without intervention; rare splenic rupture represents the most serious systemic G-CSF risk. Across the broader regenerative literature, including stromal vascular fraction preparations and pooled safety analyses, intra-articular cell therapies have shown a generally favorable short- to mid-term safety profile, although heterogeneous reporting and the potential for procedure-related complications warrant continued vigilance [28,29,30,31,32,33].

## 4. Discussion

### 4.1. Summary of Findings

This systematic review provides one of the first systematic syntheses to appraise clinical evidence for all three principal stem cell sources for IA injection therapy in KOA within a single, uniformly applied eligibility framework that additionally incorporates peripheral blood-derived therapy. Building on earlier pairwise and network meta-analyses of culture-based products, its contribution lies in the breadth and uniformity of this framing rather than in priority of comparison. The key findings are: (1) ADMSCs and BMMSCs are both supported by statistically significant pooled evidence for pain reduction at 6 months with moderate- and low-certainty evidence, respectively; (2) PBSCs demonstrate only preliminary, hypothesis-generating signals and cannot be formally pooled due to insufficient high-quality RCT evidence; (3) no direct head-to-head comparison between the three sources is available, so comparative superiority of any single source cannot be established; and (4) all sources carry a favorable safety profile with no confirmed serious treatment-related adverse events across >1200 participants.

### 4.2. Interpretation in Context

Our ADMSC pooled pain SMD (−1.23) is consistent with the 2025 Hohmann updated meta-analysis [11], which similarly identified significant pain reduction for adipose-derived stromal cells without proportionate functional improvement. The BMMSC SMD (−1.09) aligns with prior pairwise meta-analytic estimates. The addition of PBSC evidence to a systematic review is novel; while not formally poolable, the PBSC within-group improvements—particularly the 24-month structural and arthroscopic improvement reported by Saw et al.—provide a clinical signal that justifies dedicated RCT investment. The 2022 Zhu and Fu systematic review of PBSC studies identified seven studies reporting improvement but noted heterogeneous protocols and an insufficient RCT base [12]. Muthu et al. identified ADMSCs as producing more consistent efficacy than BMMSCs at the systematic review level [13], and Sadri et al. demonstrated that allogeneic ADMSCs may provide non-inferior outcomes compared to autologous preparations [14], an important finding for scalability and regulatory standardization.

### 4.3. Biological Heterogeneity and Product Classification

A central interpretive caution concerns the biological and manufacturing heterogeneity of the products grouped under each cell source. The adipose category combined culture-expanded MSCs with minimally manipulated stromal vascular fraction and micro-fragmented adipose tissue, and the bone marrow category combined culture-expanded MSCs with bone marrow aspirate concentrate. These products differ in nucleated cell composition, MSC content, immunomodulatory capacity, and regulatory classification, since culture-expanded cells are generally regulated as advanced cell therapy products whereas minimally manipulated preparations often fall under tissue or point-of-care frameworks. Pooling them may inflate between-study heterogeneity, which is consistent with the substantial I-squared values observed (62 to 70 percent), and the exploratory subgroup analysis was underpowered to resolve this. Pooled estimates for each source should therefore be read as source-level summaries across mixed product classes rather than as estimates for a single standardized cell product.

The peripheral blood source warrants separate consideration. The included PBSC studies used heterogeneous products, including G-CSF-mobilized progenitor cells collected by apheresis or venipuncture and non-mobilized peripheral blood mononuclear cell concentrates. These products are dominated by CD34-positive hematopoietic progenitors and mononuclear cells, their cellular identity and mechanism of action remain poorly characterized, and they do not consistently meet ISCT minimal MSC criteria. For these reasons, PBSC therapy is more appropriately classified as a distinct regenerative cell therapy than as a third MSC source, and its comparison with culture-based MSC products should be regarded as exploratory.

Clinical heterogeneity in the target condition is a further limitation specific to the peripheral blood evidence. Several PBSC studies enrolled younger patients with focal cartilage defects treated alongside cartilage repair or subchondral procedures, a clinical entity that differs from degenerative KOA in pathophysiology, treatment objective, and expected outcome. As shown in the sensitivity analysis, excluding these studies left only two small KOA-specific reports, so any apparent PBSC signal is confounded by indication and concomitant surgery and cannot be attributed to the cell product alone.

### 4.4. Mechanistic Considerations Across Cell Sources

The three sources differ in ways that plausibly shape clinical response. Adipose tissue yields a high frequency of stromal progenitors per unit volume and a secretome rich in anti-inflammatory and pro-angiogenic mediators, and adipose-derived cells appear comparatively resistant to donor age and senescence, which may favor consistency in an older osteoarthritic population [34,35,36,37,38,39,40,41]. Bone marrow-derived preparations contain a lower progenitor frequency that declines further with age and metabolic comorbidity, and bone marrow aspirate concentrate in particular delivers a heterogeneous mixture in which the nucleated cell and platelet fractions, rather than a defined stromal cell dose, may account for much of the effect [42,43,44,45,46,47,48]. Peripheral blood products are dominated by hematopoietic and circulating progenitor populations with angiogenic and chemotactic activity but limited classical chondrogenic potential, so their proposed mechanism is better understood as paracrine and reparative support of an existing repair response, frequently in the setting of a marrow-stimulation or cartilage repair procedure, than as direct stromal engraftment. Across all sources, the dominant mechanism is now understood to be paracrine and immunomodulatory rather than durable engraftment [49,50,51] and direct differentiation, which is consistent with the symptomatic benefit and the absence of confirmed hyaline regeneration observed in this review. Beyond cartilage, this paracrine activity may also act on the subchondral bone compartment, where osteoclast-driven remodeling through the RANKL/RANK/NFATc1 axis contributes to disease progression and is itself a tractable therapeutic target [18]; modulation of this axis represents one plausible route by which intra-articular cell products could influence the whole osteochondral unit rather than cartilage alone.

### 4.5. Comparison with Previous Reviews and the Contribution of This Study

Prior syntheses reached differing conclusions largely because of differences in scope and method. Hohmann and colleagues pooled adipose and bone marrow stromal cells and reported significant pain reduction without proportionate functional gain [11], a pattern that our source-specific estimates reproduce. Muthu and colleagues concluded that adipose-derived cells produce more consistent efficacy than bone marrow-derived cells [13], but their inclusion was restricted largely to culture-expanded products and therefore did not capture the minimally manipulated preparations that dominate point-of-care practice. Zhu and Fu confined their review to peripheral blood products and to cartilage injury rather than degenerative osteoarthritis [12], which explains both their more optimistic structural narrative and the limited transferability of their conclusions to an osteoarthritic population. The recent network meta-analysis by Xie and colleagues compared doses and tissue sources for culture-based products [6] but did not incorporate peripheral blood-derived therapies. Against this background, the specific contribution of the present review is to appraise all three sources under a single, uniformly applied eligibility framework, to separate culture-expanded from minimally manipulated products, to treat peripheral blood-derived therapy as a biologically distinct category rather than a third stromal cell source, and to grade certainty of evidence separately for each source. This integrated and deliberately conservative framing, rather than any single new effect estimate, is the principal advance offered here.

### 4.6. Clinical Translation and Implementation

Translating these findings into practice requires attention to factors that efficacy estimates alone do not capture. Autologous culture-expanded products achieve a defined cell dose but require a manufacturing facility, weeks of lead time, and classification as an advanced therapy medicinal product, whereas minimally manipulated preparations such as stromal vascular fraction, micro-fragmented adipose tissue, and bone marrow aspirate concentrate can be produced at the point of care but deliver an undefined and variable cell content, with a non-trivial dose–response relationship for bone marrow concentrate [15]. Allogeneic products offer scalability and standardization and appear to provide non-inferior early outcomes [14], which may ultimately matter more for population-level access than marginal differences in mean effect. Patient selection is also unresolved, because most included trials enrolled Kellgren–Lawrence grade II to III disease, leaving the value of these therapies in early or end-stage osteoarthritis poorly defined. Until potency assays aligned with the 2025 ISCT framework [16] allow products to be specified by function rather than by tissue of origin, source selection in clinical practice will continue to be governed as much by regulatory status, cost, and logistics as by comparative efficacy.

### 4.7. Future Directions

Several concrete steps would move the field beyond the current evidence. First, adequately powered three-arm randomized trials comparing adipose, bone marrow, and peripheral blood-derived therapies against a common comparator are needed to create a connected evidence network and to permit a valid network meta-analysis. To make this recommendation operational, such trials should pre-specify a between-group minimal clinically important difference as the target effect rather than the larger within-source SMDs reported here: for a WOMAC or VAS pain endpoint, detecting a conservative between-group standardized difference of approximately 0.4 with 80% power at a two-sided alpha of 0.05 requires on the order of 100 evaluable patients per arm (roughly 300 for a three-arm design), and a smaller anticipated difference of 0.3 would require approximately 175 per arm; allowing for 15–20% attrition over a 24-month horizon, enrolment targets of about 350–630 participants are realistic planning figures. A validated patient-reported pain or function instrument (for example WOMAC or KOOS) should serve as the single primary endpoint, with a quantitative structural measure (T2 or T1-rho mapping) and safety as key secondary endpoints, and trials should be powered on the primary endpoint while reporting all three. These figures are indicative planning estimates intended to guide design rather than definitive requirements. Second, products should be characterized prospectively using mechanism-aligned potency assays consistent with the 2025 ISCT consensus [16] and reported with sufficient manufacturing detail to allow culture-expanded and minimally manipulated preparations to be analyzed separately. In other musculoskeletal disorders, transcriptomic subtyping has begun to define biologically distinct phenotypes that respond differently to the same therapy, enabling a precision-medicine approach to treatment selection [17]; an analogous effort to phenotype both the recipient joint and the cell product would help explain the heterogeneity observed here and move the field from tissue-of-origin labels toward function-defined therapies. Third, trials should adopt a core outcome set and report quantitative structural endpoints, such as T2 and T1-rho mapping and validated semi-quantitative MRI scoring, rather than relying on symptom scores alone, so that structural modification can be distinguished from symptomatic benefit. Fourth, a minimum follow-up of 24 months and prospective registries are required to establish durability and long-term safety. Addressing these priorities would convert the within-source efficacy signals identified here into the comparative, mechanism-anchored evidence that rational source selection ultimately requires.

### 4.8. Limitations

The following limitations merit explicit acknowledgment. First, substantial I^2^ values for ADMSC (62%) and BMMSC (70%) primary analyses reflect unexplained between-study heterogeneity attributable to the combination of culture-expanded and minimally manipulated products within each source (stromal vascular fraction, micro-fragmented adipose tissue, and bone marrow aspirate concentrate), dose heterogeneity, comparator diversity, and patient population differences. Second, the PBSC evidence base (n = 5 studies, only 1 RCT) is critically insufficient for formal pooling, limiting comparative conclusions. Third, predominantly 12-month follow-up precludes conclusions about long-term durability and safety. Fourth, all source-to-source comparisons are indirect; no published trial enrolled patients in parallel stem cell source arms. Fifth, publication bias cannot be fully excluded given the relatively small number of studies per source available for pooled analysis. Only the 6-month ADMSC VAS analysis reached the ten-study threshold at which funnel-plot and Egger-test assessment is informative; for that analysis a contour-enhanced funnel plot and the Egger regression test for small-study effects are presented in Supplementary Figure S1, whereas all other pooled analyses contained too few studies for a reliable small-study-effects assessment. Sixth, the inclusion of focal cartilage defect studies, predominantly within the PBSC source, introduced clinical heterogeneity in the target population, because these patients differ from those with degenerative KOA and frequently underwent concomitant cartilage repair or subchondral procedures. Seventh, the PBSC comparison is further constrained because peripheral blood products do not uniformly meet established MSC criteria and represent a biologically distinct intervention. Eighth, we did not perform a network meta-analysis, so no formal indirect comparison of the three sources is provided; given the sparse and weakly connected evidence network, in particular, the single peripheral blood randomized trial, such an analysis would not currently yield reliable estimates. Ninth, sources of heterogeneity such as cell dose, preparation method, and comparator type were explored only through a small number of pre-specified, underpowered subgroups; the number of studies per source was insufficient to support meta-regression, so a substantial portion of the observed I^2^ remains formally unexplained and the subgroup observations should be regarded as exploratory rather than as adjusted estimates.

## 5. Conclusions

This systematic review confirms that IA injection of ADMSCs and BMMSCs provides statistically significant pain reduction in KOA, with point estimates that reach commonly cited minimal clinically important difference thresholds. These two judgements are distinct: the magnitude of effect is large enough to be clinically relevant, whereas the certainty that the true effect approximates this estimate is graded separately as moderate for ADMSCs and low for BMMSCs. The low certainty for BMMSCs, driven by substantial heterogeneity (I^2^ = 70%) and the indirectness of the comparison, means the size and even the direction of the pooled effect may change with further evidence, and the “clinically meaningful” descriptor should be read as conditional on that certainty rather than as an established treatment recommendation. PBSC therapy demonstrates promising early clinical and structural imaging signals but requires dedicated high-quality RCTs before comparative conclusions can be drawn. Head-to-head comparative trials incorporating all three sources, standardized cell characterization, longer follow-up, and objective cartilage biomarkers represent the most important methodological advancement needed to enable evidence-based source selection for patients with KOA. Because no trial compared the three sources directly, this review establishes within-source efficacy but does not support the comparative superiority of any single source, and structural findings are described as structural modification rather than confirmed cartilage regeneration.

## Figures and Tables

**Table 1:** Characteristics and outcomes of included ADMSC studies (n = 14).

Study	Year	Design	N (Active/Total)	Age (yr)	Female (%)	KL Grade	Preparation	Dose	F/U	VAS Change	WOMAC Change	SAE
Kim et al.	2023	Phase III RCT	70/140	58.3	68	II–III	Cultured auto.	1 × 10^8^	12 mo	−24.1 ± 21.3	−16.3 ± 18.1	None
Pers et al.	2016	Phase I/II	18/18	64.2	83	III	Cultured allo.	2/10/50 × 10^6^	6 mo	−25.3 ± 18.7	−14.2 ± 12.3	None
Jo et al.	2014	Pilot RCT	12/18	58.1	58	II–III	SVF auto.	1 × 10^8^/5 × 10^7^	6 mo	−18.7 ± 14.2	−13.8 ± 9.7	None
Sadri et al.	2023	Phase II RCT	20/40	54.7	62	II–III	Cultured allo.	4 × 10^7^	12 mo	−22.4 ± 16.3	−11.9 ± 8.4	None
Ulivi et al.	2023	RCT	28/56	62.1	71	II–III	mFAT + arthroscopy	N/A	12 mo	−19.8 ± 17.6	−14.7 ± 11.2	1 effusion
Kuah et al.	2018	Phase I	10/30	56.8	60	II–III	Allo. (Progenza)	1/2/6 × 10^6^	12 mo	−17.3 ± 13.9	−9.8 ± 7.6	None
Lana et al.	2021	RCT	20/40	59.3	66	II–III	SVF auto.	5 × 10^7^	6 mo	−21.2 ± 15.8	−12.4 ± 9.1	None

ADMSC, adipose-derived mesenchymal stem cell; allo., allogeneic; auto., autologous; F/U, follow-up; KL, Kellgren–Lawrence; mFAT, micro-fragmented adipose tissue; mo, months; RCT, randomized controlled trial; SAE, serious adverse event; SVF, stromal vascular fraction; VAS, visual analog scale (0–100); WOMAC, Western Ontario and McMaster Universities Osteoarthritis Index.

**Table 2:** Characteristics and outcomes of included BMMSC studies (n = 12).

Study	Year	Design	N (Active/Total)	Age (yr)	Female (%)	KL Grade	Preparation	Dose	Comparator	F/U	VAS Change	WOMAC Change	SAE
Vega et al.	2015	Phase I/II RCT	15/30	52.3	67	II–III	Cultured allo.	4 × 10^7^	HA	12 mo	−32.4 ± 22.1	−18.7 ± 14.3	None
Lamo-Espinosa	2016	Phase I/II	20/30	60.1	73	II–III	Cultured auto.	1 × 10^7^/1 × 10^8^	PRP	12 mo	−26.1 ± 19.8	−14.2 ± 11.6	None
Lee BW et al.	2025	RCT	37/74	63.4	69	II–III	Cultured auto.	1 × 10^8^	Placebo	12 mo	−23.7 ± 18.4	−13.4 ± 10.2	2 mild eff.
Davatchi et al.	2016	Phase I	4/4	59.7	50	III–IV	Cultured auto.	8–10 × 10^6^	None	6 mo	−27.0	N/A	None
Garay et al.	2021	RCT	24/48	57.8	71	II–III	BMAC auto.	~4 × 10^7^	HA	12 mo	−20.4 ± 15.1	−11.8 ± 9.4	None
Hernigou et al.	2013	Cohort	30/60	64.1	54	III	BMAC auto.	~3.5 × 10^7^	HA + OP	24 mo	−22.1 ± 17.6	−12.6 ± 10.1	None

BMAC, bone marrow aspirate concentrate; BMMSC, bone marrow-derived mesenchymal stem cell; eff., effusion; F/U, follow-up; HA, hyaluronic acid; KL, Kellgren-Lawrence; mo, months; OP, concomitant osteotomy; PRP, platelet-rich plasma; RCT, randomized controlled trial; SAE, serious adverse event; VAS, visual analog scale (0–100); WOMAC, Western Ontario and McMaster Universities Osteoarthritis Index.

**Table 3:** Characteristics and outcomes of included PBSC studies (n = 5).

Study	Year	Design	N (Active/Total)	Age (yr)	Female (%)	Indication	G-CSF Mobilization	Collection	Comparator	F/U	Primary Outcome	Key Finding	SAE
Saw et al.	2011	Prospective	5/5	41.3	40	Cartilage defect	300 µg/day × 5 d	Venipuncture	None	12 mo	Arthroscopy	Improved cartilage fill on arthroscopy	Bone pain 80%
Saw et al.	2013	RCT	25/50	34.2	44	Cartilage defect	300 µg/day × 5 d	Apheresis	HA alone	24 mo	KOOS, arthroscopy	KOOS improvement; superior arthroscopic grade vs. HA (*p* < 0.05)	Bone pain 84%
Papadopoulos	2021	Prospective	12/12	52.7	58	KOA (KL I–III)	None (non-mobilized)	Centrifuge PBMNC	None	12 mo	VAS, WOMAC	VAS −18.3; WOMAC −11.4 (within-group)	None
Yokota et al.	2019	Pilot	8/8	38.4	38	Cartilage defect	400 µg/day × 5 d	Apheresis	None	12 mo	KOOS, MRI	KOOS improved; T2 cartilage normalization at 6 mo	Bone pain 75%
Turajane et al.	2017	Prospective	20/20	53.1	65	KOA (KL II–III)	None (non-mobilized)	Centrifuge	PRP	24 mo	KOOS, MRI	Superior KOOS vs. PRP at 12 mo; T2 improved	None

G-CSF, granulocyte colony-stimulating factor; F/U, follow-up; HA, hyaluronic acid; KL, Kellgren–Lawrence; KOA, knee osteoarthritis; KOOS, Knee injury and Osteoarthritis Outcome Score; MRI, magnetic resonance imaging; PBMNC, peripheral blood mononuclear cell; PBSC, peripheral blood-derived stem cell; PRP, platelet-rich plasma; RCT, randomized controlled trial; SAE, serious adverse event; VAS, visual analog scale.

**Table 4:** Risk of bias assessment for included studies (RoB 2 for RCTs; ROBINS-I for prospective studies).

Study	Source	Design	Randomization	Alloc. Conc.	Blinding (Part.)	Blinding (Outcome)	Incomplete Data	Select. Reporting	Overall RoB
Kim 2023	ADMSC	RCT	Low	Low	Low	Low	Low	Low	Low
Pers 2016	ADMSC	Phase I/II	Low	Some concerns	Some concerns	Low	Low	Low	Some concerns
Jo 2014	ADMSC	Pilot RCT	Low	Low	Some concerns	Low	Low	Low	Some concerns
Sadri 2023	ADMSC	Phase II RCT	Low	Low	Low	Low	Low	Low	Low
Ulivi 2023	ADMSC	RCT	Low	Low	Some concerns	Low	Low	Low	Some concerns
Vega 2015	BMMSC	Phase I/II RCT	Low	Low	Some concerns	Low	Low	Low	Some concerns
Lee BW 2025	BMMSC	RCT	Low	Low	Low	Low	Low	Low	Low
Lamo-Esp. 2016	BMMSC	Phase I/II	Low	Some concerns	High	Some concerns	Low	Low	High
Hernigou 2013	BMMSC	Cohort	N/A	N/A	High	High	Some concerns	Some concerns	High
Saw 2013	PBSC	RCT	Low	Some concerns	High	Some concerns	Low	Low	Some concerns
Papadopoulos 2021	PBSC	Prospective	N/A	N/A	High	Some concerns	Low	Low	High
Turajane 2017	PBSC	Prospective	N/A	N/A	Some concerns	Some concerns	Low	Low	High

N/A, not applicable for the randomization and allocation-concealment domains, which do not exist within the ROBINS-I framework and are, therefore, left blank for non-randomized studies; the corresponding seven ROBINS-I domains for these studies are reported in full in Table 5. Color coding: green = low risk of bias; yellow = some concerns; red = high risk of bias. Alloc. Conc., allocation concealment; part., participants.

**Table 5:** Domain-level ROBINS-I risk-of-bias assessment for the included non-randomized studies.

Study	D1 Confounding	D2 Participant Selection	D3 Intervention Classification	D4 Deviations	D5 Missing Data	D6 Outcome Measurement	D7 Reported Result	Overall
**Hernigou 2013 (BMMSC)**	Serious	Moderate	Low	Moderate	Moderate	Serious	Low	**Serious**
**Papadopoulos 2021 (PBSC)**	Serious	Moderate	Low	Moderate	Low	Serious	Low	**Serious**
**Turajane 2017 (PBSC)**	Serious	Moderate	Low	Moderate	Low	Serious	Moderate	**Serious**

ROBINS-I overall judgements use the standard categories (Low, Moderate, Serious, Critical, No information); the term “Serious” corresponds to the “High” overall rating shown in Table 4. D1–D7 denote the seven ROBINS-I domains: confounding; selection of participants into the study; classification of interventions; deviations from intended interventions; missing data; measurement of outcomes; and selection of the reported result. Confounding and outcome-measurement domains were the principal drivers of the overall judgements, reflecting non-randomized allocation, frequent concomitant procedures, and open-label assessment of subjective endpoints. BMMSC, bone marrow-derived mesenchymal stem cell; PBSC, peripheral blood-derived stem cell.

**Table 6:** Summary of pooled effect sizes by stem cell source and outcome (random-effects model, DerSimonian–Laird).

Cell Source	Outcome	Studies (k)	Participants (n)	Pooled SMD (95% CI)	I^2^ (%)	*p*-Value	GRADE
ADMSC	VAS (6 mo)	10	548	−1.23 (−1.61 to −0.85)	62	<0.001	Moderate
ADMSC	WOMAC total (6 mo)	8	467	−0.81 (−1.14 to −0.48)	65	<0.001	Moderate
ADMSC	VAS (12 mo)	7	412	−1.18 (−1.57 to −0.79)	60	<0.001	Moderate
BMMSC	VAS (6 mo)	8	392	−1.09 (−1.55 to −0.63)	70	<0.001	Low
BMMSC	WOMAC total (6 mo)	6	334	−0.63 (−1.02 to −0.24)	69	0.001	Low
BMMSC	VAS (12 mo)	5	278	−1.04 (−1.49 to −0.59)	68	<0.001	Low
PBSC	VAS (6–12 mo)	3	148	Not pooled †	N/A	N/A	Very low
PBSC	WOMAC (12 mo)	2	88	Not pooled (high heterog.)	N/A	N/A	Very low

CI, confidence interval; GRADE, Grading of Recommendations, Assessment, Development, and Evaluations; I^2^, measure of statistical heterogeneity; k, number of studies; mo, months; n, number of participants; SMD, standardized mean difference (Hedges’ g); VAS, visual analog scale; WOMAC, Western Ontario and McMaster Universities Osteoarthritis Index. † Peripheral blood-derived stem cell outcomes were not statistically pooled, consistent with Section 3.4.3; substantial heterogeneity in mobilization protocols and outcome instruments and the availability of only a single randomized trial precluded a valid pooled estimate. Values are, therefore, reported descriptively in the text rather than as a meta-analytic summary.

## Data Availability

No new data were created or analyzed in this study. Data are available in the individual studies cited in the reference list.

## Supplementary Materials

The following supporting information can be downloaded at: https://www.mdpi.com/article/10.3390/bioengineering13070771/s1, Supplementary File S1. Full Electronic Search Strategy; Supplementary Figure S1. The contour-enhanced funnel plot and Egger’s regression test for the only pooled analysis that reached the ten-study threshold (6-month ADMSC pain, k = 10).
