# Supplementary material for: Comparative Efficacy and Safety of Intra-Articular Adipose-Derived, Bone Marrow-Derived, and Peripheral Blood-Derived Stem Cell Injections for Knee Osteoarthritis: A Systematic Review
Source: Bioengineering (Basel). 2026 Jul 1;13(7):771. doi: 10.3390/bioengineering13070771 (PMC13405813; doi:10.3390/bioengineering13070771)

bioengineering

MDPI

Supplementary File S1. Full Electronic Search Strategy

Manuscript: Comparative Efficacy and Safety of Intra-articular Adipose-, Bone Marrow-, and Peripheral Blood-derived Stem Cell Injections for Knee Osteoarthritis: A Systematic Review.

Databases: MEDLINE (via PubMed), Embase, Cochrane CENTRAL, and Scopus. Trial registries: ClinicalTrials.gov and WHO ICTRP. Coverage: database inception to 31 December 2025 (the search was updated through 31 December 2025 during revision, applying identical eligibility criteria). No language filter was applied at the search stage; non-English reports were excluded at screening. The strategy combined three concept blocks with the Boolean operator AND: (1) stem cell source, (2) route of administration, and (3) target condition.

1. MEDLINE (PubMed) — primary strategy

#1 Stem cell source

```
("adipose-derived stem cell*"[tiab] OR "adipose derived stem cell*"[tiab] OR
"adipose-derived mesenchymal"[tiab] OR ADSC[tiab] OR ADMSC[tiab] OR
"stromal vascular fraction"[tiab] OR SVF[tiab] OR "micro-fragmented adipose"[tiab] OR
"microfragmented adipose"[tiab] OR mFAT[tiab] OR "bone marrow mesenchymal"[tiab] OR
"bone marrow-derived"[tiab] OR BMMSC[tiab] OR BMSC[tiab] OR
"bone marrow aspirate concentrate"[tiab] OR BMAC[tiab] OR "bone marrow concentrate"[tiab] OR
"peripheral blood stem cell*"[tiab] OR "peripheral blood-derived"[tiab] OR
"peripheral blood mononuclear"[tiab] OR PBMNC[tiab] OR PBSC[tiab] OR CD34[tiab] OR
"mesenchymal stem cell*"[tiab] OR "mesenchymal stromal cell*"[tiab] OR
"Mesenchymal Stem Cells"[Mesh] OR "Adipose Tissue"[Mesh:NoExp])
```

#2 Route of administration

```
("intra-articular"[tiab] OR intraarticular[tiab] OR "intra articular"[tiab] OR
injection*[tiab] OR "Injections, Intra-Articular"[Mesh])
```

#3 Target condition

```
("knee osteoarthritis"[tiab] OR "osteoarthritis of the knee"[tiab] OR "knee OA"[tiab] OR
gonarthrosis[tiab] OR "cartilage defect*"[tiab] OR "chondral defect*"[tiab] OR
"Osteoarthritis, Knee"[Mesh] OR ("Osteoarthritis"[Mesh] AND "Knee Joint"[Mesh]))
```

#4 Combination

```
#1 AND #2 AND #3
```

Filters applied after the combined search: Humans[Mesh]; publication date from inception to 2025/12/31. Animal-only and in-vitro records were removed at screening.

2. Embase (Elsevier) — Emtree adaptation

```
#1  'mesenchymal stem cell'/exp OR 'adipose derived stem cell'/exp OR
```

```
    'stromal vascular fraction':ab,ti OR 'bone marrow aspirate concentrate':ab,ti OR
    'peripheral blood stem cell'/exp OR (admsc OR bmmsc OR bmac OR pbsc OR svf):ab,ti
#2  'intraarticular drug administration'/exp OR 'intra-articular':ab,ti OR injection*:ab,ti
#3  'knee osteoarthritis'/exp OR 'knee osteoarthritis':ab,ti OR gonarthrosis:ab,ti OR
    'cartilage defect':ab,ti
#4  #1 AND #2 AND #3
```

3. Cochrane CENTRAL — adaptation

```
#1  (MeSH descriptor: [Mesenchymal Stem Cells] explode all trees)
#2  ("adipose-derived" OR "bone marrow" OR "peripheral blood" OR mesenchymal OR
    SVF OR BMAC OR ADMSC OR BMMSC OR PBSC):ti,ab,kw
#3  ("intra-articular" OR intraarticular OR injection*):ti,ab,kw
#4  (MeSH descriptor: [Osteoarthritis, Knee] explode all trees)
#5  ("knee osteoarthritis" OR gonarthrosis OR "cartilage defect*"):ti,ab,kw
#6  (#1 OR #2) AND #3 AND (#4 OR #5)
```

4. Scopus — adaptation

```
TITLE-ABS-KEY(("adipose-derived stem cell*" OR "stromal vascular fraction" OR
"bone marrow aspirate concentrate" OR "peripheral blood stem cell*" OR
"mesenchymal stem cell*" OR admsc OR bmmsc OR bmac OR pbsc OR svf)
AND ("intra-articular" OR intraarticular OR injection*)
AND ("knee osteoarthritis" OR gonarthrosis OR "cartilage defect*"))
```

Note: Search strings were syntactically adapted to each database’s controlled vocabulary and field tags while preserving the same three-block logic. Records from all sources were de-duplicated in EndNote and screened in Rayyan against the PICOS criteria reported in the main text.

bioengineering

Supplementary Figure S1 — the contour-enhanced funnel plot and Egger's regression test for the only pooled analysis that reached the ten-study threshold (6-month ADMSC pain, k = 10).

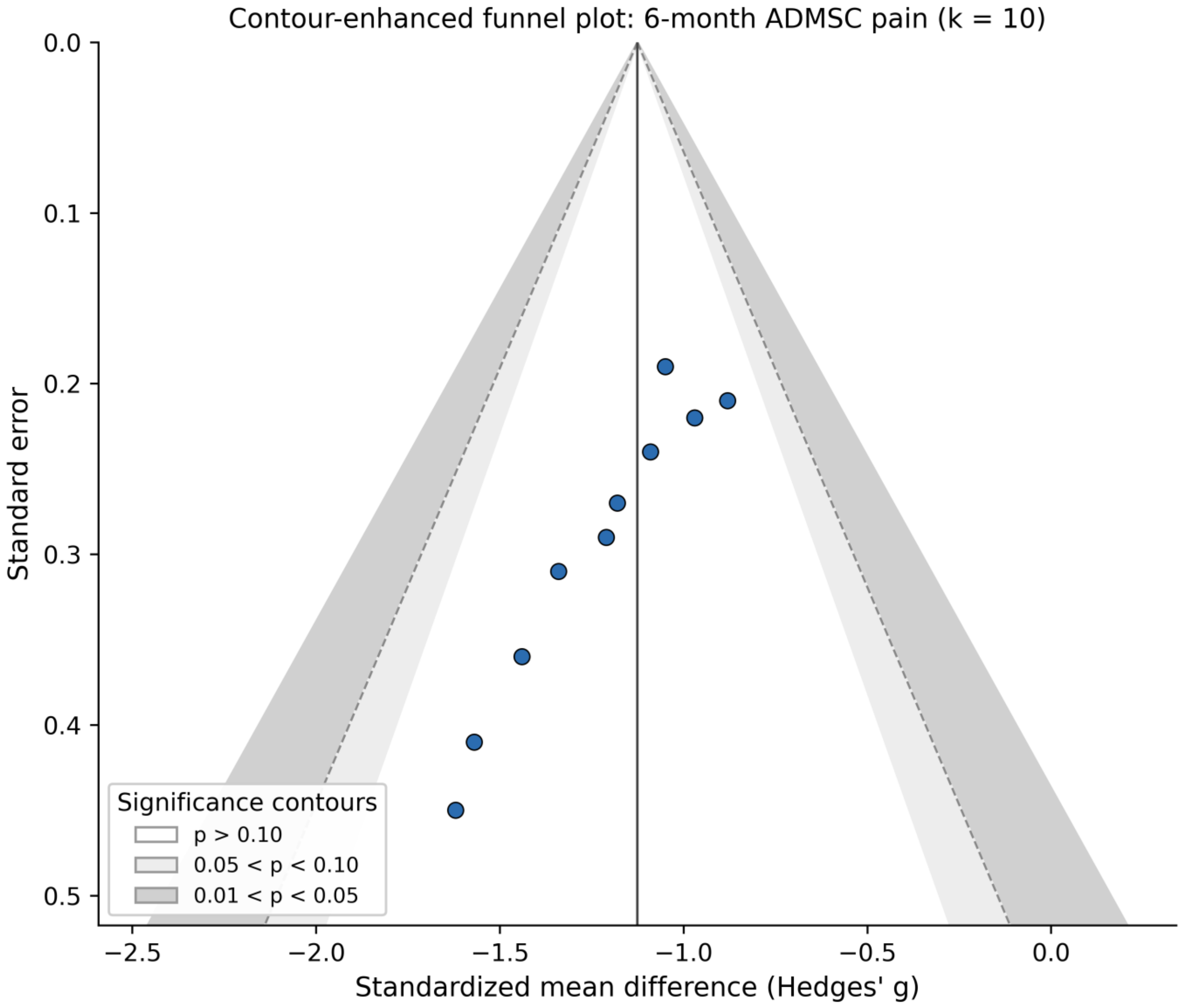

Supplement: Supplementary file 1 [file bioengineering-13-00771-s001.zip › bioengineering-4389035-supplementary.pdf]
